# A Pilot Study on the Satisfaction of Long-Term Care Services in Taiwan

**DOI:** 10.3390/ijerph19010090

**Published:** 2021-12-22

**Authors:** Amy H. I. Lee, He-Yau Kang, Yu-Ai Liu

**Affiliations:** 1 Department of Industrial Management, Chung Hua University, Hsinchu 300, Taiwan; amylee@chu.edu.tw; 2 Department of Industrial Engineering and Management, National Chin-Yi University of Technology, Taichung 411, Taiwan; qwedog13579@gmail.com

**Keywords:** long-term care, structural equation modeling, artificial neural network, satisfactory services, consistency reliability

## Abstract

For many developed countries and regions, long-term care is becoming an important issue due to demographic changes and an increasing willingness and need of family members to let the elderly be taken care of by non-family members. Thus, effectively managing long-term care needs has become a major societal concern. In this paper, the public attitude towards long-term care and the satisfaction of long-term care services in Taiwan are examined. First, internal consistency reliability, exploratory factor analysis (EFA) and confirmatory factor analysis (CFA) are applied to delete unimportant indicators. Second, structural equation modeling (SEM) is used to determine which indicators have a statistically significant influence on the public attitude toward long-term care and on the satisfaction of long-term care services. Third, artificial neural network (ANN) is applied to understand the relative importance of the indicators in influencing the public attitude and satisfaction of long-term care services. The contribution of this study is significant because some of the factors investigated in the study should be stressed by the government or institutions to provide more satisfactory services to the elderly and their families.

## 1. Introduction

Long-term care includes both medical and non-medical care and can be provided at home, in assisted living facilities or in nursing homes. While traditional medical care is concerned with cure and recovery, long-term medical care attempts to alleviate suffering, maintain the best possible quality of life, reduce discomfort, improve the limitations caused by diseases and disability, and maintain the best possible levels of functioning [1]. Non-medical care provides support services for activities of daily living, including dressing, bathing, using the bathroom, getting in and out of bed, etc. Long-term care service is often needed by people with developmental disabilities and mental illnesses, chronic diseases, injuries, disabling conditions, physiological frailty, and dementia [2]. Since these conditions often associate with and increase exponentially with age, the rapid growth of aging people is creating a greater need for long-term care services. Older persons are more vulnerable to chronic and disabling diseases and loss of cognitive capacity, and, therefore, are more prone to the loss of personal autonomy, medical supports, financial independence, and social supports [3].

Long-term care has become a critical societal issue. From the perspective of the government, how to provide sufficient long-term care services in a rapidly aging population is becoming a major societal issue. From the perspective of the elderly and family members, how to select the most appropriate type of long-term care service to meet the needs and the situations of the elderly and family members is a difficult and important decision to be made. From the perspective of long-term care service providers, how to provide good long-term care services to meet the various needs of the elderly and family members is essential not only to keep the institutions sustainable but also to meet the humane principles of healthcare. By learning the needs, expectations, perceptions, and satisfaction regarding the services that long-term care recipients and their family members receive, the long-term care service providers can identify improvement directions, both in hardware facilities and in software services.

In Taiwan, society is aging fast due to declining fertility rate and increasing life expectancy. As the number of young people staying in the workforce keeps increasing, all-day-long family care of elderly is becoming unattainable, and good quality and affordable long-term care services are in need. Government and long-term care service providers play crucial roles in supporting and providing the services. This study, therefore, aims to learn the factors that influence the public attitude toward long-term care services and the satisfaction of the long-term care services in Taiwan. A model that integrates structural equation modeling (SEM) and artificial neural network (ANN) is proposed in this research to fulfill the task.

SEM is a good tool to test the hypothesized relationship, but it can only examine linear models and it may sometimes oversimplify the complexities of relationships among the factors [4,5,6]. On the other hand, ANN can be used to examine the complex linear and non-linear relationships. However, while ANN has a good performance in prediction, it cannot test the hypothesized relationship [6,7]. A bibliometric analysis performed by Zabukovšek et al. [8] showed that the integration of SEM and ANN is gaining importance in various research fields and is becoming a promising approach. This research, therefore, integrates SEM with ANN to better understand the factors that influence the public attitude and satisfaction of long-term care services in Taiwan.

The structure of this paper is organized as follows. Literature related to consumer satisfaction and quality of healthcare services is reviewed, and information regarding demographic conditions and long-term care services in Taiwan is presented in Section 2. A SEM-ANN model for examining the long-term care service problem is proposed in Section 3. A case study is performed in Section 4. Conclusions and remarks are made in the final section.

## 2. Literature Review

### 2.1. Consumer Satisfaction and Quality of Healthcare Services

Healthcare service quality is defined as the implementation of medical science and technology to maximize the health benefit without correspondingly increasing the risk [9]. Many providers have shifted from providing healthcare services based the preferences and decisions of medical professionals to services shaped by the views and needs of the users [10,11]. Thus, the views of healthcare recipients has become important for healthcare service providers to monitor, manage, and improve their service quality, and healthcare is expected to be delivered by skilled professional providers to satisfy customer expectations and needs. However, healthcare service quality is difficult to measure due to the variety of care service processes and the different kinds of interactions between patients and providers, including service characteristics and ethical considerations in the healthcare sector [12].

Satisfaction of patients is influenced by factors that may be rather different from the consumption of normal goods. The factors include: the attitudes of nurses toward patients; the capacity to deliver prompt service without wasting time; the ability to disseminate information to patients; the availability of up-to-date equipment; the hospital’s ability to render 24 h service; the patience of the doctor to clearly explain the condition of patients and provide detail information about their medication; and attractiveness and cleanliness of the hospital [11,13]. The assessment of the satisfaction and perceived service quality of patients is an important issue for healthcare providers, helping them to achieve the following: to learn what the patients need; to understand where, when, and how service should be provided; to know what possible improvements can be made; and to best distribute the scarce resources of the healthcare service [11].

An emerging quality assessment framework divides customers’ experience value into extrinsic and intrinsic values [14,15]. Such a framework may be more suitable for healthcare services because these services involve both emotional and social dimensions [15]. The extrinsic value further contains utilitarian value and extrinsic social value. The utilitarian value, or functional value, considers factors such as how effective a treatment is and how clean the facilities are [15]. The extrinsic social value considers aspects such as how pleasant the interactions with the healthcare personnel are. The intrinsic value can be segregated into emotional value, epistemic value, and intrinsic social value [15]. The emotional value, which can be either active or reactive, is defined as the utility from mood or emotional states [16]. For example, it can be the empathy received from health personnel and not being ignored by the personnel [15]. The epistemic value is derived from customer’s knowledge, beliefs and information [15]. The intrinsic social value captures the utility recognized by social groups, such as whether the patient is being treated respectfully [15].

Greater consumer participation in the planning, delivery, and evaluation of healthcare can improve the quality and appropriateness of healthcare [17]. Consumers are the primary decision makers who choose the healthcare service, and they can be care recipients of their family members. Measuring satisfaction in long-term care has become important so that healthcare services can better meet individual client needs and be responsive [18]. Flexibility, consumer autonomy, and quality of life are some important factors for higher consumer satisfaction and have been used to select long-term care facilities [18]. 

Service quality is defined as the provider’s ability to assess, confirm and meet the needs of clients [19]. A well-known multi-dimensional research instrument to capture consumer expectations and perceptions of a service is SERVQUAL, proposed by Parasuraman, Zeithaml, and Berry [20]. Under the SERVQUAL, there are five dimensions: tangibles; reliability; responsiveness; assurance; and empathy [20]. SERVQUAL has been used in some studies in healthcare [21,22,23,24,25]. Some scholars, however, stated that the SERVQUAL was not originally designed for healthcare facilities and might miss important contextual aspects of patient experiences, such as experiences with physicians and nurses [15,26]. In addition, since the SERVQUAL assesses both individual’s expectations and perceptions of actual performance at the same time, it may introduce bias [26]. A long survey may increase respondent burden [26]. Some models have been developed based on the SERVQUAL and other concepts to fit the healthcare service industry. While the SERVQUAL model, which was first developed by Parasuraman et al. [20], aims to compare the differences between the customer’s expectations before and after the service delivery, the SERVPERF model, which was later proposed by Cronin and Taylor [27], focuses on performance measures based on the customer’s perceptions. That is, the SERVPERF model discards the expectation € component of the SERVQUAL and instead uses a performance (P) component. A set of healthcare service quality (HEALTHQUAL) measurement items was developed by Lee [12] for modern healthcare services from three perspectives: the patient, the accreditation agency and the hospital. Under the proposed model, two dimensions were considered: care process and results. Under the care process dimension, there were four components: empathy; tangible; safety; and efficiency. Under the results dimension, the component was the degree of improvements of care service.

Multi-criteria decision-making techniques have been adopted in a broad range of areas in the healthcare, with the top area being disease diagnosis and treatment, followed by priority setting, health technology assessment, and formulary management. For example, Hsu and Dai [28] proposed a model to evaluate the outsourcing of nursing care attendants in the healthcare sector. A modified Delphi technique was applied first to select the most appropriate evaluation criteria, and the AHP was adopted next to calculate the weights of the criteria and to rank the nursing care attendant vendors. Wang et al. [29] proposed a two-step model to determine the ranking of the criteria for quality of care provided by long-term care institutions in Taiwan. In the first step, the five constructs of SERVQUAL were adopted, and various criteria under each construct were listed based on the opinions of experts. The fuzzy Delphi method (FDM) was applied next to extract the most important criteria under each construct. In the second step, the fuzzy analytic hierarchy process (FAHP) was used to synthesize the opinions of experts and family members of long-term care institutions, and the importance and the ranking of the criteria for quality of care provided by long-term care institutions were obtained. Büyüközkan et al. [30] proposed a service quality evaluation framework by adopting the FAHP methodology. Through a literature survey, especially on SERVQUAL, and expert views, the authors identified criteria for service quality evaluation in the healthcare industry and then constructed an evaluation hierarchy for FAHP analysis. The proposed framework was applied to evaluate the healthcare sector in Turkey. Lee and Kang [31] studied a senior daycare center location evaluation problem. A network with criteria under benefits, opportunities, costs, and risk aspects of locations was constructed, and the interrelationships among the criteria determined using fuzzy interpretative structural modeling. Fuzzy analytic network process was adopted to calculate the importance weights of the criteria and the ranking of the daycare center locations.

### 2.2. Long-Term Care Services

Long-term care systems can be divergent in different countries and regions due to culture, demographic conditions, government support, etc. Brodsky and Clarfield [32] studied the long-term care conditions in some industrialized and developing countries and regions. Several aspects were examined, including: the nature of entitlements, targeting and finance; service delivery strategies; and issues of integration between long-term care and health and social services. The authors concluded that although most industrialized countries and regions offered long-term care, the level and mix of the service varied among countries and regions because of the policies, targeting, entitlement and finance. Freeman et al. [33] examined long-term care organizations and financing. Demographic changes and the increasing need of long-term care in various countries and regions were introduced. Both informal care and formal care provisions were presented. Funding approaches of long-term care, basically including out-of-pocket payments, private insurance, tax-based support, and social insurance, were analyzed. Stallard [2] studied the long-term care for aging populations in the USA. The results of some long-term care surveys were analyzed, long-term care disabilities were estimated, long-term care demographics were presented, and the uses of home and community-based long-term care services were introduced. Crawford et al. [34] studied long-term care spending and hospital use among the older population in England between 2009 and 2018 after the reductions in government funding for long-term care on public hospitals. Ariaans et al. [35] provided a comprehensive overview of long-term cares in OECD countries and regions and proposed an updated long-term care typology. Six method-driven types were identified: residual public system; private supply system; public supply system; evolving public supply system; need-based supply system; and evolving private need-based system. Nine content-driven clusters, each containing one to five countries and regions with strong ties, were distinguished.

Due to declining fertility rates and increasing life expectancy, many developing and developed countries and regions are facing aging populations, and Taiwan is no exception. Taiwanese society is aging fast due to a declining fertility rate and increasing life expectancy, and it entered an aged society (populations with more than 14% elderly with age of 65 or higher) in 2018 and is expected to enter a super-aged society (populations with more than 20% elderly with age of 65 or higher) in 2025 [36]. 

The average life expectancy in Taiwan was 81.3 years in 2020, with 78.1 years for men and 84.7 years for women [37]. Many baby boomers, who were born between the end of World War II and the 1960s, have been growing into the elderly population after 2010. In addition, the dropping of the birth rate is causing a dramatic decrease in the population aged under 14 years. While the United Nations sets a threshold for fertility rate (children born per woman) of 1.3 as “ultra-low,” the rate was only 1.07 children per woman in Taiwan in 2020, ranked last out of the 227 countries and regions studied [38]. Young people in Taiwan are more and more reluctant to marry and have children; some reasons for this include increasing housing prices, stagnant salaries, and growing income inequality [39]. As a consequence, the total dependency ratio will increase from 40.2% to most likely 102% by 2070, with 18% of child dependency ratio and 84% of elderly dependency ratio [36]. This means that every 100 persons of working age will need to support 102 people by 2070, and 82.35% (=84%/102%) of the support will provided to the elderly. Such demographic changes in Taiwan will become a great burden to society in the future because of the increasing demand of supporting elderly people and decrease in productivity for the reduced workforce. 

Because of strong family ties in Chinese culture, the burden of long-term care has been rested on family members. However, the birth rate is continuing to decline, and the number of women staying in the workforce keeps increasing. Globalization has also contributed to increasing number of young people working overseas. Even though many families rely on foreign caregivers, the government has laws that limit the employment of foreign caregivers, and the expertise and quality of the eldercare provided by the foreign caregivers may not be satisfactory. These all make family care in danger of losing its function in the near future. Thus, the need for long-term care services has increased substantially, and it will skyrocket in the very near future. High-quality, affordable and accessible long-term care services are needed to allow the elderly to receive care with dignity, and a compassionate society that respects and cares for all of its senior citizens can be fostered [40].

The government in Taiwan began implementing a 10-year long-term care plan in 2007 (TLTCP 1.0) to solve the aging society problem. Since the implementation of the 10-year long-term care plan, the total number of institutions that provide various kinds of services and the total number of people who receive different care services has increased. The Long-term Care Services Act was enacted on 3 June 2015 as the principal legal basis for developing comprehensive long-term care services in Taiwan [41]. The act aimed to integrate the related acts of long-term care services and to construct a long-term care network in order to construct a better and higher-quality service delivery system [42]. Nevertheless, there were many challenges in quality, quantity, distribution, integration, and efficiency issues in the long-term care system. The major problems of the long-term care plan included inadequate funding, insufficient home and community care services, insufficient care personnel, and a gap between resources allocated to urban and rural areas [43]. Wang et al. [44] used the database of TLTCP 1.0 to examine different patterns of use of home- and community-based services of the elderly and to understand the effects of the different use patterns on the use of institutional long-term care services.

To counteract the problems of the long-term care plan, a 10-year long-term care plan 2.0 (TLTCP 2.0) was passed by the Executive Yuan on 29 September 2016. The new long-term care plan aims to create a localized, community-based long-term care model that integrates social care, medical care, and preventive health resources [40]. The plan establishes a three-tier system, comprising community-based integrated service centers, combination day care and service centers, and long-term care stations in alleys and lanes [40]. In essence, the new plan retains the long-term care services provided under the previous 10-year plan, but extends the service to more people, develops an integrated community long term care system, and strengthens the electronic management system [42]; that is, the new plan expands both the number of people eligible for assistance and the kinds of aid available [45]. The new coverage includes people over the age of 50 with dementia, members of indigenous tribes from low-lying areas over 55 who suffer from disabilities, the mentally or physically challenged and those suffering from disabilities under 49, and infirm senior citizens over 65 [40]. A more detailed description of and challenges faced by the long-term care policy process in Taiwan can be found in Hsu and Chen [46].

Even though the government has implemented a 10-year long-term care plan starting from 2007 and a 10-year long-term care plan 2.0 starting from 2017, long-term care is basically a self-pay service for the majority of the elderly and their families. However, with the rapid change in demographics and soaring healthcare costs, out-of-pocket long-term care services may be a heavy burden for many of them. Therefore, how to select the most appropriate long-term care service based on the physical condition of the elderly and the situation of the family is an important decision many families need to make, soon or sometime in the future. Long-term care service providers also need to understand and measure their service quality and to learn the satisfaction of the long-term care recipients and their family members so that better and more focused quality service can be provided. The government should also understand the need of people to devise regulations and provide support to long-term care recipients. Whether government support has a significant impact on the public attitude and satisfaction regarding long-term care services should be understood. With a good understanding of these issues, the government can develop appropriate programs to promote long-term care services. 

## 3. Data and Methods

### 3.1. Sampling and Data Collection

Based on the literature review and current long-term care condition in Taiwan, the authors aim to understand the factors that influence the public attitude and satisfaction levels regarding long-term care services in Taiwan. A survey questionnaire was applied to examine the hypotheses regarding public attitude and satisfaction levels. A preliminary questionnaire was designed and was pretested with two university professors and eight experts in the long-term care field. Some revisions and adjustment of the questionnaire were made to make it more understandable and relevant. The subjects included adults who themselves or whose family members need any kind of long-term care services. A convenience sample approach was used. The questionnaire was designed and distributed through Google Forms, Facebook and Line. Professors and students of several universities in Taiwan were asked to fill out the questionnaire and to pass the questionnaire to their friends and relatives. The questionnaire was also given out to the management and workers in long-term care service institutions.Respondents answered the questionnaire either independently or with help from their relatives. For elderly people who were able to answer the questions but could not use computers or high-tech devices, their relatives could help them fulfill the task. The relatives of the elderly could also fill out the questionnaire based on their observations of the services received by the elderly. Out of 521 questionnaires collected, 413 usable questionnaires were yielded. A complete information of the respondents and the data collected will be presented in Section 4. 

### 3.2. Variable Measurement

For the first part of the conceptual model, the public attitude toward long-term care (D) is the dependent variable (construct), and government support (A), long-term care service providers (B), and services received by elderly and family (C) are the independent variables (constructs). For the second part of the conceptual model, the satisfaction of long-term care services (S) is the dependent variable (construct), and government support (A), long-term care service providers (B), services received by elderly and family (C), and public attitude toward long-term care (D) are the independent variables (constructs). There are multiple indicators under each construct, and they are measured by respondents on a 7-point Likert with scale “1” (strongly disagree) to “7” (strongly agree). The constructs and indicators (after deletion of three indicators discussed later in the case study) are as shown in Table 1. The control variables include gender, age, and education level. 

### 3.3. Method of Analysis: SEM-ANN Approach

An integrated SEM and ANN approach is used to better understand the factors that influence public attitude toward long-term care and the factors that influence public satisfaction of long-term care services. There are five steps, as follows.

**Figure 1:**
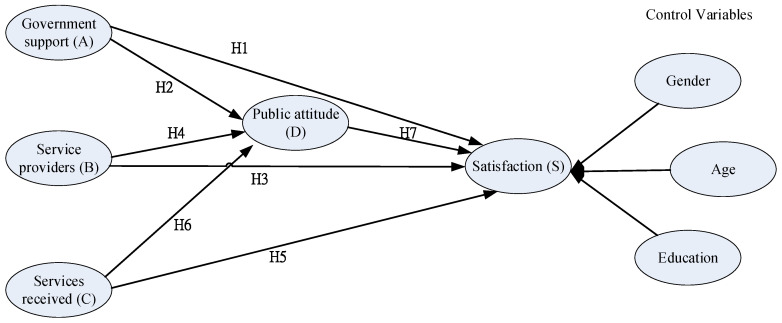
Conceptual model.

Step 1. Construct a conceptual model. As shown in Figure 1, the constructs include: government support (A), long-term care service providers (B), services received by elderly and family (C), public attitude toward long-term care (D), and satisfaction of long-term care services (S). The hypotheses are as follows:

**Hypothesis** **1** **(H1).** *Government support (A)has a significant and positive association with satisfaction of long-term care services (S)*.

**Hypothesis** **2** **(H2).** *Government support (A)has a significant and positive association with public attitude toward long-term care (D)*.

**Hypothesis** **3** **(H3).** *Long-term care service providers (B)has a significant and positive association with satisfaction of long-term care services (S)*.

**Hypothesis** **4** **(H4).** *Long-term care service providers (B)has a significant and positive association with public attitude toward long-term care (D)*.

**Hypothesis** **5** **(H5).** *Services received by elderly and family (C)has a significant and positive association with satisfaction of long-term care services (S)*.

**Hypothesis** **6** **(H6).** *Services received by elderly and family (C)has a significant and positive association with public attitude toward long-term care (D)*.

**Hypothesis** **7** **(H7).** *Public attitude toward long-term care (D)has a significant and positive association with satisfaction of long-term care services (S)*.

Step 2. Prepare a questionnaire. The questionnaire based on the conceptual model is pretested, and a more relevant and understandable questionnaire is generated after the required revisions are made. The revised questionnaire is given out to respondents to fill out.

Step 3. Carry out statistical data analysis. Statistical data analysis, including internal consistency reliability, exploratory factor analysis (EFA) and confirmatory factor analysis (CFA), is performed. The internal consistency reliability of each construct can be measured by Cronbach’s *α* coefficient. The EFA is carried out to determine data patterns, analyze relationships and decrease data. The CFA is applied to examine the reliability, convergent validity, and discriminant validity. The indicators that do not meet assessment requirements are deleted from the model for further analysis.

Step 4. Perform structural equation modeling (SEM). After the statistical analysis, SEM is adopted to assess the measurement model and to perform the hypotheses testing [47,48,49,50,51]. The SEM is a regression-based multivariate statistical analysis technique used to analyze structural relationships among observed variables and unobserved variables and to test the data fit of models formulated a priori [52,53]. Software package SPSS Amos 21 is used to perform the SEM, and the hypotheses in Figure 1 are examined.

Step 5. Perform artificial neural network (ANN) [54,55,56,57]. Due to the fact that a simple linear model may not fully capture the complexity of a problem and an artificial intelligence (AI) approach could tackle a non-linear regression model effectively [58], ANN, a popular AI technique is applied next based on the results from the SEM. The multi-layer perceptron (MLP) with a feed-forward back-propagation (FFBP) algorithm is applied. The MLP consists of three types of layers: an input layer, an output layer, and at least one hidden layer. The input layer receives the signal to be processed, and the output layer is the outcome of the stimuli. The hidden layer is the computational engine and maps the relations between the output layer and input layer [59]. In the FFBP, the input signals are fed in forward direction whereas the errors are propagated in backward direction [58]. The software package SPSS Statistics 22 is used to solve the ANN.

## 4. Case Study

A preliminary questionnaire was designed based on questionnaire contents of past related works and interviews with experts in the long-term care field. As stated before, the questionnaire was pretested, and some revisions of the content were made to make it more relevant and understandable. The questionnaire distributed in this research is divided into two parts: the first part is the basic information of the respondents and the second part is the content related to the subject of this research. The first part includes questions regarding gender, age and education level. The second part of the questionnaire includes 39 questions (indicators) under five constructs, and 7-point Likert scale, ranging from strongly disagree (1) to strongly agree (7), is used to collect respondents’ opinions. A total of 521 questionnaires were collected, and 413 valid questionnaires were generated. 

### 4.1. Statistical Data Analysis

Table 2 shows the respondents’ profile for the first part of the questionnaire. The internal consistency reliability of each construct can be measured by Cronbach’s *α* coefficient, which ranges from zero (unreliable) to one (perfect reliability), and a value of 0.70 or greater is considered acceptable [60]. In this case, all Cronbach’s *α* were greater than 0.9; therefore, all indicators were kept in for further analysis.

The collected responses were analyzed using both exploratory factor analysis (EFA) and confirmatory factor analysis (CFA). The EFA was carried out first, and the rotated component matrix was prepared. The extraction method was principal component analysis. The rotation method was varimax with Kaiser normalization, and the rotation converged in six iterations. The value of Kaiser Meyer Olkin (KMO) was 0.929, higher than the marvelous value of 0.9 [61]. The significance value under the Bartlett’s test of sphericity was 0.000, and it was under 0.05, which is a 95% confidence level of significance. In this study, indicators with a loading of less than 0.55 to the construct were deleted. In the end, three indicators were deleted, and the final rotated component matrix is shown in Table 3. As shown before, Table 1 shows the constructs and indicators after the analysis.

The CFA was carried out next to examine the reliability, convergent validity, and discriminant validity, and the results are shown in Table 4, Table 5, Table 6, Table 7. Table 4 shows the psychometric properties of the instrument, including mean, standard deviation (*SD*), loadings, and reliability for each indicator and Cronbach’s *α*, composite reliability (*CR*), and average variance extracted (*AVE*) for each construct. By ensuring that *CR* and Cronbach’s α exceed 0.7, adequate reliability for confirmatory purposes can be met [60]. For convergent validity, all indicator loadings should exceed 0.70, and *AVE* for each construct should exceed 0.5 [62,63]. Among the 36 indicators, 35 indicators had loadings exceeding 0.7, except one (S6) with loadings of 0.691, which still surpassed the acceptable level of 0.5 [64,65]. Since all *AVE*s were greater than 0.5, this indicates that each construct could explain more than half of the variance of its indicators on average. The bootstrap critical ratios showed that all indicators were statistically significant at *p* < 0.001. As a result, all indicators were retained for further analysis.

Discriminant validity between constructs can be assessed by examining whether the square root of *AVE* for each construct is higher than its correlation with any other latent variable [62]. Table 5 shows the intercorrelatons of the constructs. The diagonal values are the square root of *AVE*, which should be higher than the non-diagonal elements in the same row or column. Since no values of non-diagonal elements in the same row or column items were larger than the value of the diagonal element, the discriminant validity was supported. Discriminant validity can also be examined by heterotrait-monotrait ratio of correlations (HTMT). The result of HTMT, in which all the values were less than the conservative threshold of 0.85 [66], is shown in Table 6. The discriminant validity was also supported. 

**Figure 2:**
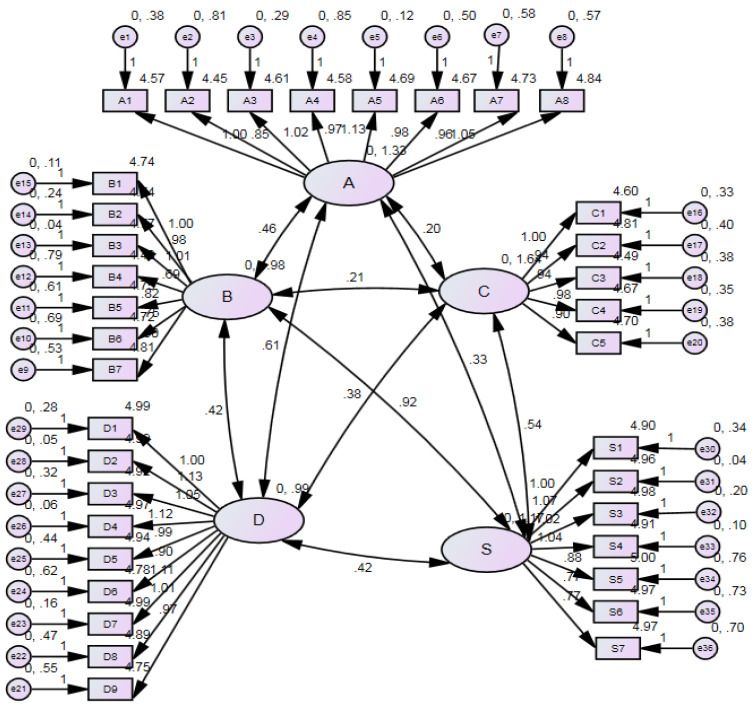
Path diagram from the CFA.

Table 7 shows the estimates of the CFA model. Each indicator to its construct had a *p*-value of less than 0.001. This indicates that the regression weight of each indicator is greater than zero. The path diagram from the CFA is as depicted in Figure 2. A part of the figure is interpreted here. The regression weights of A1 and A2 to A are 1.00 and 0.85, respectively. The intercepts of A1 and A2 are 4.57 and 4.45, respectively. The variance of A is 1.33, and the variance of e1 is 0.38. The covariance between A and B is 0.46. The goodness of fit of the CFA model can be estimated by four common measures: the ratio of *χ*^*2*^ statistics to the degree of freedom (df); normed fit index (NFI); comparative fit index (CFI); and the root mean square error of approximation (RMSEA) [67]. The recommended and acceptable values for the four measures are listed in Table 8 [68,69,70,71,72,73,74]. In this CFA model, the ratio of *χ*^*2*^ statistics to the degree of freedom (df) was 4.674, the NFI was 0.864, the CFI was 0.889, and the RMSEA was 0.094. Even though some of the goodness-of-fit indicators were not within the recommended threshold limits, they were still acceptable. Since all indicators met the satisfactory requirements, they were used in the model for the SEM. 

### 4.2. Structural Equation Modeling (SEM)

**Figure 3:**
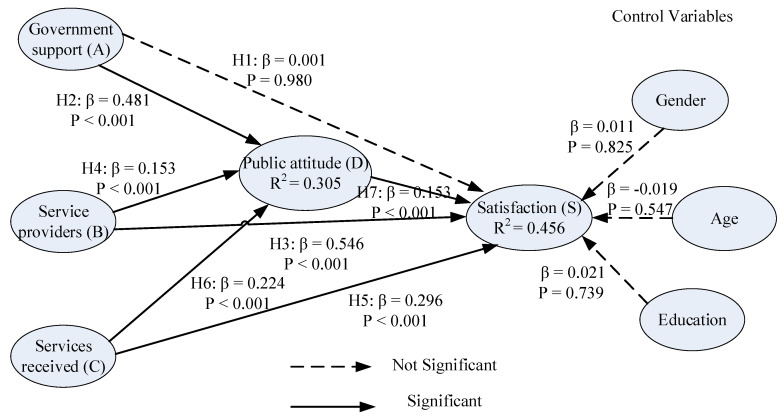
Results for the structural model.

The SEM was implemented to test the hypotheses. The measurement model of the CFA was converted into the SEM by replacing the bi-directional arrows with single-headed arrows [59]. The structural model was examined using the four measures applied in the CFA: the ratio of *χ*^*2*^ statistics to the degree of freedom (df); normed fit index (NFI); comparative fit index (CFI); and the root mean square error of approximation (RMSEA). In this SEM model, the ratio of *χ*^*2*^ statistics to the degree of freedom (df) was 4.723, NFI was 0.861, CFI was 0.887, and RMSEA was 0.095, as shown in Table 9. The results show that the proposed structural model was an acceptable model fit to the data. The significance of each hypotheses and the variance explained for each latent endogenous variable are shown in Figure 3. The *R^2^*, indicating the explanatory power or variance explained of the latent endogenous variable, was obtained for each regression equation. With *R^2^* for all endogenous variables exceeding 0.26, as suggested by Cohen [75], the predictive power of the model was sound. Table 10 shows the significant testing results of the structural model. Government support (A) has a significant effect on the public attitude toward long-term care (D) (*β* = 0.48, critical ratio = 10.054, *p* < 0.001); thus, hypothesis H2 is confirmed. Both H4 and H6 are also confirmed with long-term care service providers (B) having a significant effect on the public attitude toward long-term care (D) (*β* = 0.153, critical ratio = 3.467, *p *< 0.001) and services received by elderly and family (C) having a significant effect on the public attitude toward long-term care (D) (*β* = 0.224, critical ratio = 5.085, *p* < 0.001). The result indicates that government support has stronger impact on the public attitude toward long-term care than the other two constructs. Therefore, the government should provide greater long-term care support to improve public attitude toward the service. On the other hand, government support (A) does not have a significant effect on the satisfaction of long-term care services (S) (*β* = 0.001, critical ratio = 0.025, *p* = 0.980), and hypothesis H1 is rejected. Hypotheses H3, H5 and H7 are confirmed. Based on the result of H3, long-term care service providers (B) have the most significant effect on the satisfaction of long-term care services (S) (*β* = 0.546, critical ratio = 12.759, *p* < 0.001). Based on the result of H5, services received by elderly and family (C) also have a significant effect on the satisfaction of long-term care services (S) (*β *= 0.296, critical ratio = 7.179, *p* < 0.001). Based on the result of H7, public attitude toward long-term care (D) has a significant effect on the satisfaction of long-term care services (S) (*β* = 0.153, critical ratio = 3.330, *p* < 0.001). In addition, gender, age and education background do not have significant effects on the satisfaction of long-term care services (S).

### 4.3. Artificial Neural Network (ANN)

**Figure 4:**
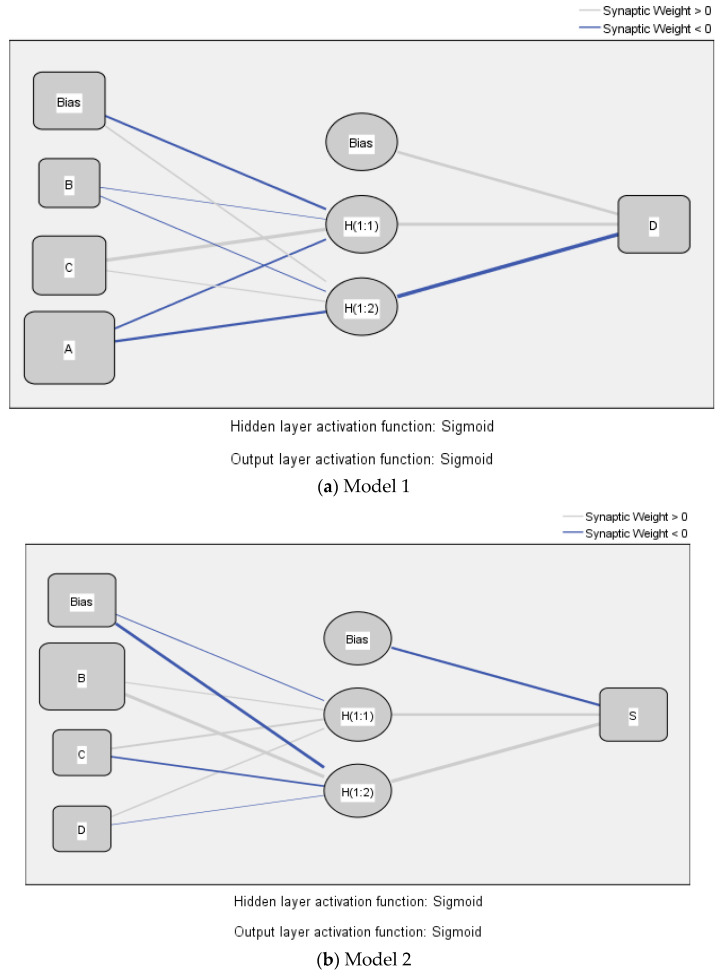
ANN models.

Since simple linear models may not be sufficient to capture the complexity of actual world problems, an AI approach may provide relatively sophisticated non-linear regression models with greater accuracy [58]. In this research, ANN models were developed using the statistically significant predictors from the SEM. The feed-forward back-propagation (FFBP) multi-layer perceptron (MLP) was applied, and sigmoid function was used as the activation function for both the hidden layer and the output layer. Based on the results from the SEM in Figure 3, two ANN models were decomposed, as shown in Figure 4. Model 1 has one output (public attitude toward long-term care: D) and three inputs: government support (A), long-term care service providers (B), and services received by elderly and family (C). Model 2 has one output (satisfaction of long-term care services: S) and three inputs: long-term care service providers (B), services received by elderly and family (C), and public attitude toward long-term care (D). Each model has a hidden layer, and the number of neurons are generated automatically. Model 1 has two neurons, so does model 2. To avoid the over-fitting problem, a 10-fold cross-validation procedure was applied. In this case, 90% of the data was used for network training, and 10% of the data was used for testing [8,58,67]. The predictive accuracy of the ANNs can be measured by root mean square of error (RMSE) [76]. Table 11 shows the RMSE values for both training and testing data sets of the two ANN models. The RMSE values were small, and the mean values of the RMSE with 10 runs in model 1 for the training set and for the testing set were 0.112 and 0.104, respectively. Those in model 2 for the training set and for the testing set were 0.103 and 0.094, respectively. The results indicate an accurate and reliable prediction of the models in capturing the relationships between predictors and outputs.

A sensitivity analysis was carried out next to understand the relative importance of inputs in predicting the output value. The relative importance of each input in predicting the output for 10 runs in each model is shown in Table 12. The average relative importance of each input for the 10 runs was calculated next. The normalized importance of each input is calculated as the ratio of the average relative importance of each input over the maximum average relative importance, expressed in percentage form [5,58]. The relative strengths of the causal relationships between inputs and the output are examined based on the normalized importance [67]. According to Table 12, for model 1, the most significant predictor of public attitude toward long-term care (D) was government support (A), followed by services received by elderly and family (C) and long-term care service providers (B). As a predictor of public attitude toward long-term care (D), government support (A) was found to be almost twice more significant than services received by elderly and family (C) and three times more significant than long-term care service providers (B). Therefore, the government should provide sufficient support to improve public attitude. In model 2, the order of importance towards the satisfaction of long-term care services (S) in descending order was long-term care service providers (B), public attitude toward long-term care (D) and services received by elderly and family (C). As a predictor of satisfaction of long-term care services (S), long-term care service providers (B) was found to be almost three times more significant than public attitude toward long-term care (D) and services received by elderly and family (C). This implies that long-term care service providers play a crucial role in satisfying long-term care recipients and their family members. 

Results of the ANN are similar to those form the SEM, but some differences are found. Under the SEM, as depicted in Figure 3, services received by elderly (C) has a higher significant effect than public attitude toward long-term care (D) on the satisfaction of long-term care services (S). On the other hand, under the ANN, as shown in Table 12, public attitude toward long-term care (D) has a higher significant effect than services received by elderly (C) on the satisfaction of long-term care services (S), even though the difference in the importance of the two is not very large.

## 5. Conclusions

This study aims to provide insights in understanding the public’s attitude toward long-term care and the satisfaction of long-term care services in Taiwan. A model that incorporates structural equation modelling (SEM) and artificial neural network (ANN) is proposed to solve the problem. While the SEM is useful for performing hypothesis analysis, the utilization of the ANN can provide additional verification of the results generated by the SEM analysis. In addition, the ANN can capture both linear and nonlinear relationships between endogenous and exogenous variables and has a good performance in prediction. Even though the results from the SEM and ANN are quite similar, there are still some differences in the ranking of constructs. The ANN is also known to perform a good prediction of future behavior.

The results of the SEM found that government support has the most significant effect on the public attitude toward long-term care, followed by long-term care service providers and services received by elderly and family. In addition, long-term care service providers have the most significant effect on the satisfaction of long-term care services, followed by services received by elderly and family and public attitude towards long-term care. Results of the ANN basically confirmed the findings of the SEM. However, some minor differences were discovered. While services received by elderly has a higher significant effect on the satisfaction of long-term care services than public attitude towards long-term care under the SEM, public attitude towards long-term care was found to have a higher significant effect on the satisfaction of long-term care services than services received by elderly under the ANN. Nevertheless, the difference in the importance of the two is not very large under the ANN.

The findings of this research provide contributions to the decision-making process of the government and long-term care service institutions. The study found that government support has stronger impact on the public attitude toward long-term care than services received by elderly and family and long-term care service providers. Based on these results, the government can understand the perception of the public towards government regulations and support for long-term care and what kind of regulations, support, and programs the government should devise to improve the long-term care conditions in Taiwan. In addition, long-term care service providers can understand what kind of hardware facilities the elderly need and what kind of services they demand the most in long-term care service institutions. As a result, the service providers can construct well-equipped institutions and provide good-quality services to the elderly. 

## Figures and Tables

**Table 1:** Constructs and indicators.

Constructs	Indicators
A. Government support	A1. The government implements good regulations toward long-term care institutions.
	A2. The government needs to formulate more regulations for long-term care institutions.
	A3. The government provides comprehensive support to long-term care recipients.
	A4. The government should provide more funding for long-term care-related services.
	A5. The government should promote long-term care insurance.
	A6. The government should provide more community-based long-term care services.
	A7. Providing government subsidies to long-term care recipients is the best way for caring them.
	A8. The government should promote more long-term care related programs.
B. Long-term care service providers	B1. Long-term care institutions charge reasonable fees.
	B2. The charging standards of long-term care institutions do not need to be unified.
	B3. Long-term care institutions need to arrange staff training and education in a timely basis.
	B4. Long-term care institutions have the right to choose the person to be cared for.
	B5. Long-term care institutions need to provide a family meeting space for family members when visiting the care recipients.
	B6. Long-term care institutions need to provide different care methods for different care recipients.
	B7. The environment of long-term care institutions is very important.
C. Services received by elderly and family	C1. Group activities should be held for the elderly regularly.
	C2. Outdoor activities should be provided for the elderly in a timely and regular manner.
	C3. Single room can be provided for the elderly if preferred.
	C4. Senior daycare centers need to provide transportation services.
	C5. Violating the regulations of caring for the elderly should be fined strictly.
D. Public attitude toward long-term care	D1. I understand the long-term care plan 2.0.
	D2. If I need to choose a long-term care institution, I will choose a suburban area.
	D3. I think the elderly care resources in Taiwan are sufficient at present.
	D4. I agree with the government’s promotion of community-based long-term care services.
	D5. I think the current long-term care workforce is sufficient.
	D6. I think the government can afford the financial expenditures of the long-term care support.
	D7. I think it is inconvenient to use long-term care resources in remote areas.
	D8. I am willing to enter long-term care related industries.
	D9. If one day I need long-term care, I will choose home care because my home is more familiar and comfortable.
S. Satisfaction of long-term care services	S1. I am satisfied with the fees charged by the general long-term care institutions.
	S2. I am satisfied with the government’s long-term care subsidies received by the long-term care institutions.
	S3. I am satisfied with Taiwan’s current long-term care policy.
	S4. I am satisfied with the locations of current long-term care institutions in Taiwan.
	S5. I am satisfied with Taiwan’s current long-term care services.
	S6. I am satisfied with the environment of current long-term care institutions in Taiwan.
	S7. I am satisfied with current services received by long-term care recipients in Taiwan.

**Table 2:** Profile of respondents (*N* = 413).

Characteristics	Categories	Number of Responses	Percentage
Gender	Male	207	50.12%
	Female	206	49.88%
Age	Below 20	17	4.12%
	20–29	131	31.72%
	30–39	26	6.30%
	40–49	98	23.73%
	50–59	135	32.69%
	Over 60	6	1.45%
Education level	Elementary school	3	0.73%
	Middle school	19	4.60%
	High school	65	15.74%
	Undergraduate	271	65.62%
	Graduate	55	13.32%

**Table 3:** Rotated component matrix.

	Component				
	1	2	3	4	5
A1	0.203	0.858	0.106	0.064	0.103
A2	0.251	0.735	0.130	0.073	−0.021
A3	0.236	0.862	0.063	0.053	0.081
A4	0.292	0.757	0.112	0.139	0.001
A5	0.266	0.902	0.113	0.058	0.030
A6	0.253	0.814	0.118	0.137	0.010
A7	0.215	0.825	0.142	0.043	−0.011
A8	0.257	0.833	0.074	0.020	0.038
B1	0.132	0.126	0.881	0.295	0.019
B2	0.121	0.131	0.857	0.324	−0.018
B3	0.125	0.108	0.895	0.296	0.002
B4	0.053	0.152	0.784	0.213	0.159
B5	0.102	0.125	0.846	0.237	0.048
B6	0.127	0.125	0.793	0.271	0.070
B7	0.122	0.083	0.854	0.245	0.013
C1	0.100	0.054	0.038	0.105	0.913
C2	0.119	0.043	0.086	0.157	0.883
C3	0.141	0.051	−0.017	0.088	0.899
C4	0.143	0.019	0.040	0.113	0.900
C5	0.178	0.000	0.063	0.117	0.882
D1	0.846	0.232	0.113	0.099	0.044
D2	0.907	0.218	0.104	0.142	0.123
D3	0.837	0.272	0.131	0.161	0.110
D4	0.908	0.226	0.108	0.121	0.087
D5	0.803	0.317	0.177	0.081	0.127
D6	0.707	0.384	0.015	0.134	0.234
D7	0.882	0.226	0.097	0.151	0.121
D8	0.821	0.219	0.124	0.168	0.064
D9	0.764	0.334	0.075	0.112	0.206
S1	0.764	0.078	0.320	0.769	0.208
S2	0.235	0.111	0.289	0.844	0.263
S3	0.184	0.100	0.325	0.803	0.250
S4	0.189	0.104	0.271	0.838	0.225
S5	0.184	0.109	0.306	0.757	0.058
S6	0.118	0.049	0.465	0.710	−0.025
S7	0.113	0.068	0.447	0.722	−0.063

**Table 4:** Measurement model results.

Construct/Indicators	Mean	*SD*	Loadings	Cronbach’s *α*	*CR*	*AVE*
A. Government support				0.951	0.954	0.722
A1	4.57	1.308	0.881			
A2	4.45	1.328	0.734			
A3	4.61	1.291	0.910			
A4	4.58	1.450	0.767			
A5	4.69	1.345	0.968			
A6	4.67	1.329	0.846			
A7	4.73	1.342	0.822			
A8	4.84	1.423	0.848			
B. Long-term care service providers				0.961	0.962	0.768
B1	4.74	1.445	0.974			
B2	4.74	1.465	0.942			
B3	4.77	1.436	0.975			
B4	4.48	1.321	0.738			
B5	4.75	1.400	0.827			
B6	4.72	1.338	0.783			
B7	4.81	1.461	0.866			
C. Services received by elderly and family				0.951	0.953	0.801
C1	4.60	1.408	0.913			
C2	4.81	1.364	0.884			
C3	4.49	1.350	0.889			
C4	4.67	1.392	0.907			
C5	4.70	1.313	0.883			
D. Public attitude toward long-term care				0.962	0.966	0.762
D1	4.99	1.130	0.883			
D2	4.99	1.152	0.971			
D3	4.92	1.195	0.877			
D4	4.97	1.142	0.968			
D5	4.94	1.191	0.826			
D6	4.78	1.199	0.748			
D7	4.99	1.180	0.939			
D8	4.89	1.224	0.825			
D9	4.75	1.217	0.789			
S. Satisfaction of long-term care services				0.943	0.945	0.714
S1	4.90	1.228	0.878			
S2	4.96	1.180	0.966			
S3	4.98	1.184	0.926			
S4	4.91	1.166	0.962			
S5	5.00	1.288	0.733			
S6	4.97	1.192	0.691			
S7	4.97	1.185	0.702			

**Table 5:** Discriminant validity analysis based on Fornell-Larcker criterion.

	A	B	C	D	S
A	0.850				
B	0.283	0.876			
C	0.135	0.117	0.895		
D	0.533	0.299	0.294	0.873	
S	0.267	0.606	0.393	0.394	0.845

**Table 6:** HTMT ratios of correlations.

	A	B	C	D	S
A					
B	0.316				
C	0.113	0.177			
D	0.599	0.375	0.318		
S	0.287	0.810	0.322	0.409	

**Table 7:** Estimates of the CFA model.

Indicator	Standardized Estimate	Unstandardized Estimate	Standard Error	Critical Ratio	*p* Value
A1	0.881	1.000			
A2	0.736	0.848	0.045	18.727	***
A3	0.909	1.018	0.036	28.260	***
A4	0.770	0.969	0.048	20.156	***
A5	0.967	1.128	0.034	32.872	***
A6	0.848	0.977	0.041	24.047	***
A7	0.822	0.957	0.042	22.756	***
A8	0.848	1.047	0.043	24.177	***
B1	0.974	1.000			
B2	0.943	0.981	0.021	47.373	***
B3	0.989	1.010	0.014	72.397	***
B4	0.740	0.694	0.032	21.431	***
B5	0.828	0.823	0.029	28.101	***
B6	0.785	0.746	0.031	24.432	***
B7	0.866	0.899	0.028	32.369	***
C1	0.911	1.000			
C2	0.885	0.941	0.033	28.182	***
C3	0.889	0.935	0.033	28.595	***
C4	0.906	0.982	0.033	30.016	***
C5	0.884	0.904	0.032	27.878	***
D1	0.883	1.000			
D2	0.982	1.135	0.032	35.442	***
D3	0.879	1.053	0.040	26.407	***
D4	0.977	1.119	0.032	34.962	***
D5	0.829	0.990	0.042	23.366	***
D6	0.752	0.904	0.046	19.600	***
D7	0.940	0.966	0.045	21.482	***
D8	0.827	1.112	0.036	31.145	***
D9	0.792	1.015	0.044	23.262	***
S1	0.881	1.000			
S2	0.984	1.073	0.031	35.015	***
S3	0.927	1.016	0.034	29.961	***
S4	0.961	1.036	0.032	32.734	***
S5	0.736	0.877	0.046	18.850	***
S6	0.697	0.768	0.044	17.294	***
S7	0.705	0.773	0.044	17.631	***

*** 0.001 of significance.

**Table 8:** Goodness of fit- CFA model.

Goodness of Fit Measures	Recommended Value	Acceptable Value	CFA Model
*χ*^*2*^ statistics/df	≤3.0	≤5.0	4.674
Normed fit index (NFI)	≥0.90	≥0.85	0.864
Comparative fit index (CFI)	≥0.90	≥0.85	0.889
Root mean square error of approximation (RMSEA)	≤0.08	≤0.1	0.094

**Table 9:** Goodness of fit- structural model.

Goodness of Fit Measures	Recommended Value	Acceptable Value	Structural Model
*χ*^*2*^ statistics/df	≤3.0	≤5.0	4.723
Normed fit index (NFI)	≥0.90	≥0.85	0.861
Comparative fit index (CFI)	≥0.90	≥0.85	0.887
Root mean square error of approximation (RMSEA)	≤0.08	≤0.1	0.095

**Table 10:** Estimates of the structural model.

Hypothesis	Construct			Standardized Estimate	Unstandardized Estimate	Standard Error	Critical Ratio	*p* Value
H1	A	→	S	0.001	0.001	0.041	0.025	0.980
H2	A	→	D	0.481	0.402	0.040	10.054	***
H3	B	→	S	0.546	0.406	0.032	12.759	***
H4	B	→	D	0.153	0.105	0.030	3.467	***
H5	C	→	S	0.296	0.241	0.034	7.179	***
H6	C	→	D	0.224	0.168	0.033	5.085	***
H7	D	→	S	0.153	0.166	0.050	3.330	***

*** 0.001 of significance.

**Table 11:** RMSE values of ANNs.

Artificial Neural Networks	Model 1 Input Neurons: A, B, C Output Neuron: D		Model 2 Input Neurons: B, C, D Output Neuron: S	
	Training	Testing	Training	Testing
ANN1	0.114	0.109	0.101	0.098
ANN2	0.109	0.099	0.100	0.122
ANN3	0.117	0.120	0.104	0.092
ANN4	0.107	0.111	0.099	0.095
ANN5	0.117	0.098	0.101	0.084
ANN6	0.116	0.106	0.100	0.066
ANN7	0.106	0.112	0.109	0.095
ANN8	0.115	0.087	0.110	0.093
ANN9	0.107	0.094	0.106	0.090
ANN10	0.107	0.102	0.100	0.104
Mean	0.112	0.104	0.103	0.094
Standard deviation	0.005	0.010	0.004	0.014

Note: A = government support; B = long-term care service providers; C = services received by elderly and family; D = public attitude toward long-term care; S = satisfaction of long-term care services.

**Table 12:** Sensitivity analysis of ANNs.

Artificial Neural Networks	Model 1 Output Neuron: D			Model 2 Output Neuron: S		
	A	B	C	B	C	D
ANN1	0.575	0.178	0.246	0.579	0.207	0.214
ANN2	0.548	0.155	0.298	0.555	0.199	0.245
ANN3	0.383	0.237	0.380	0.525	0.197	0.279
ANN4	0.520	0.178	0.303	0.523	0.214	0.163
ANN5	0.604	0.177	0.219	0.68	0.162	0.158
ANN6	0.485	0.254	0.262	0.599	0.223	0.177
ANN7	0.541	0.130	0.329	0.586	0.147	0.267
ANN8	0.554	0.177	0.269	0.518	0.22	0.262
ANN9	0.530	0.170	0.300	0.614	0.174	0.212
ANN10	0.567	0.122	0.310	0.583	0.284	0.133
Average relative importance	0.531	0.178	0.292	0.576	0.203	0.211
Normalized importance (%)	100.0	33.5	54.9	100.0	35.2	36.6

Note: A = government support; B = long-term care service providers; C = services received by elderly and family; D = public attitude toward long-term care; S = satisfaction of long-term care services.

## Data Availability

The data presented in this study are available on request from the corresponding author.
